# Modulatory Effects of Jujuboside A on Amino Acid Neurotransmitter Profiles in Tic Disorder

**DOI:** 10.1002/brb3.71041

**Published:** 2025-11-11

**Authors:** Fei Fan, Si Zhang, Hongwei Wu, Meiyu Zhang, Fei Han

**Affiliations:** ^1^ Department of Paediatrics Guang'anmen Hospital China Academy of Chinese Medical Sciences Beijing China; ^2^ Suzhou TCM Hospital Affiliated to Nanjing University of Chinese Medicine Suzhou China; ^3^ Institute of Chinese Materia Medica China Academy of Chinese Medical Sciences Beijing China; ^4^ Experimental Research Center China Academy of Chinese Medical Sciences Beijing China

**Keywords:** gamma‐aminobutyric acid, glutamate, jujuboside A, tic disorder

## Abstract

**Objective:**

This study investigates the impact of jujuboside A (JuA) on the levels of glutamate (Glu) and gamma‐aminobutyric acid (GABA) in the extracellular fluid of the caudate putamen (CPu) in a rat model of tic disorder (TD). It also compares the therapeutic effects of JuA alone and in combination with Tiapride on TD.

**Method:**

The Sprague–Dawley (SD) rats were randomly assigned to five distinct groups: the normal group, the model group, the JuA group, the Tiapride group, and the JuA + Tiapride group. The TD rat model was established through the induction method utilizing intraperitoneal injection of iminodipropionitrile (IDPN). Microdialysis technology was utilized to obtain extracellular fluid from the CPu of rats in each group, and high‐performance liquid chromatography (HPLC) was employed to ascertain the levels of Glu and GABA. The stereotypical behavior scores of rats in each group were observed and recorded. Immunohistochemistry was utilized to compare the expression levels of glutamate transporter‐1(GLT‐1) and glutamate/aspartate transporter (GLAST) proteins in the CPu tissue of rats across the different groups.

**Results:**

In the TD rats, the Glu content in the CPu extracellular fluid was significantly increased, while the GABA content was significantly decreased, accompanied by a notable increase in stereotypical behavior scores. Both JuA and Tiapride reduced the Glu content and increased the GABA content in the CPu extracellular fluid of TD rats, improving stereotypical behaviors. The combination of JuA and Tiapride showed superior effects compared to either treatment alone, potentially related to the upregulation of GLT‐1 and GLAST protein expression in the CPu tissue.

**Conclusion:**

JuA can improve stereotypical behaviors in TD rats by regulating the levels of Glu and GABA in the CPu extracellular fluid, possibly through the upregulation of GLT‐1 and GLAST protein expression. The combination of JuA and Tiapride offers enhanced therapeutic effects, providing a new approach for the treatment of TD.

## Graphical abstract

Jujuboside A ameliorates tic disorder in rats by reducing striatal glutamate and increasing GABA levels, with enhanced efficacy when combined with Tiapride, potentially through upregulation of glutamate transporters GLT‐1 and GLAST.

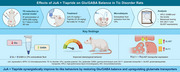

## 1 Background

There are more than 200 neurotransmitters in the human brain, and these neurotransmitters are involved in a variety of neural activities and physiological functions, such as mood and behavior (Ngernsutivorakul et al. 2018; Cools et al. 2008). Alterations in neurotransmitter levels have an impact on many central nervous system disorders, including tic disorder (TD) (Set and Warner 2021; Eissa et al. 2018; Shi et al. 2023). The quantification of alterations in neurotransmitter concentrations within brain regions across a spectrum of neuropsychiatric disorders constitutes a significant domain of scientific inquiry. At present, a limited number of methodologies possess the capability to gauge alterations in neurotransmitter concentrations over extended periods, spanning days to months; among these, microdialysis is particularly distinguished. Microdialysis boasts exceptional sensitivity and can achieve a temporal resolution of 1–5 min, this technique allows for the collection of dialysate from awake animals, followed by the application of high‐performance liquid chromatography (HPLC) to perform multiplex analysis of neurotransmitters in the brain dialysate (Hershey and Kennedy 2013; Soukupová et al. 2018). Studies have shown that the degeneration and death of dopaminergic neurons in the caudate putamen (CPu) can lead to TD, a process jointly regulated by the neurotransmitters glutamate (Glu) and gamma‐aminobutyric acid (GABA) in the central nervous system (CNS) (Héja et al. 2019; Ding et al. 2017). Jujuboside A (JuA), a biologically active compound derived from *Semen Ziziphi Spinosae*, has demonstrated neuroprotective activities via anti‐oxidative and anti‐inflammatory effects (Liu et al. 2014; Tabassum et al. 2019). Recent studies have shown that JuA has the potential to treat TD, but the specific mechanism is not yet clear (Fan, Hao et al. 2022). Tiapride is an atypical antipsychotic medicine, which is widely used in European countries for the treatment of TD (Zangani et al. 2022; Lenka and Jankovic 2024).

In order to further understand the effects of glutaminergic excitability and GABAergic neurotransmission on the onset of TD, as well as the ameliorative effects of the two drugs, Tiapride and JuA, we employed intracranial dual‐probe microdialysis technology to systematically monitor the microdialysis levels of Glu and GABA in the extracellular fluid of the CPu in a TD rat model under awake conditions. Intraperitoneal injection of iminodipropionitrile (IDPN) was employed to induce a TD rat model, which replicates the characteristics of TD in terms of both behavioral and pathological changes (Li et al. 2024; Long et al. 2019). Changes were observed in the microdialysis levels of Glu and GABA in the extracellular fluid of the CPu in awake rat models of TD during treatment with three different drug regimens: Tiapride, JuA, and a combination of Tiapride and JuA. These observed trends will enable us to propose a hypothesis concerning the neuropathological mechanisms underlying TD. Studies have previously provided detailed reviews on the advantages and limitations of microdialysis technology, comparing it to other in vivo perfusion techniques (Renno et al. 1998; Kennedy et al. 2002). The use of microdialysis technology for detecting levels of Glu and GABA in both human and animal models has been well validated (Sarlo and Holton 2021).

In conclusion, this manuscript investigates the impact of three drug regimens on the microdialysis levels of Glu and GABA in the extracellular fluid of the CPu in a rat model of TD, along with the potential mechanisms behind these effects.

## 2 Materials and Methods

### 2.1 Animals

A total of 30 SPF male Sprague‐Dawley (SD) rats (6–8 weeks old, 180–200 g)were purchased from Beijing HFK Bioscience Co., Ltd. (Animal License No: SCXK(Jing)2022‐0002). The rats were kept in the animal room of the Institute of Basic Theory, China Academy of Chinese Medical Sciences, with a 12 h light/12 h dark cycle circumstance at a constant temperature of 22 ± 2°C and a humidity of 45% ± 5%. The rats had unlimited access to standard water and food pellets ad libitum and were given 1‐week adaptation period. All experimental protocols were approved by the Animal Studies Ethics Committee of Medical Experiment Center, China Academy of Chinese Medical Sciences (approval number, ERCCACMS21‐2111‐16).

### 2.2 Drugs and Reagon Preparation

Tiapride hydrochloride tablets (100 mg/tablet) were purchased from Jiangsu Tasly Diyi Pharmaceutical Co., Ltd. (State Medical Permit No.: H3202601, Jiangsu, China), JuA (10 mg) was obtained from Beijing Solarbio Technology Co., LTD (Catalog No.: 55466‐04‐1), and 3,3’‐Iminodipropionitrile (IDPN, 25 g/bottle) was sourced from Sigma‐Aldrich Corporation (Catalog No.: 5147‐80‐8, United States). Tiapride was mixed with 0.9% NaCl to prepare a suspension solution with a concentration of 3.125 mg/mL. JuA was dissolved in 0.9% NaCl to form a suspension solution with a concentration of 1 mg/mL (Li et al. 2022). IDPN was combined with 0.9% NaCl to create a suspension solution with a concentration of 30 mg/mL. The prepared drug solutions were then sealed and stored at 4°C for subsequent use.

Glu (Lot No. 95436‐100MG), GABA (Lot No. A2129‐100MG), sodium acetate (Lot No. 71188‐250G), IDPN (Lot No. 5147‐80‐8), and ortho‐phthalaldehyde (OPA) (Lot No. BCBT6314) are from Sigma‐Aldrich, USA. Isoflurane (Lot No. 1902801) is from RWD Life Science Co., Ltd., Shenzhen, China. β‐Mercaptoethanol (Lot No. 0482‐100ML) is from Amresco, USA. Boric acid (Lot No. CN‐882320‐04) is from Acros Organics, USA. Tetrahydrofuran (Lot No. 50113) is from Mallinckrodt Baker, USA. Methanol (Lot No. F23N4P204) is from Dima Technology Co., Ltd., Beijing, China. Sodium chloride injection (Approval No. H20184083) is from Hebei Tiancheng Pharmaceutical Co., Ltd., China. Glutamate transporter‐1 (GLT‐1) antibody (Lot No. ab120066) and glutamate/aspartate transporter (GLAST) antibody (Lot No. ab181036) are from Abcam, UK.

### 2.3 Model Establishment, Animals Groups, and Treatment Methods

After a 7‐day acclimatization period, 30 male SD rats were randomly divided into a normal group (*n* = 6) and a model group (*n* = 24). The modeling procedure was performed according to the method described in the referenced literature (Diamond et al. 1982), the TD rat model was established by intraperitoneal injection of IDPN (30 mg/mL, 250 mg/kg) in the model group. Rats in the normal group were injected with an equivalent volume of normal saline instead, once daily for 7 consecutive days. Successful induction of the model was confirmed when all rats in the model group exhibited varying degrees of stereotyped behaviors (such as vertical or horizontal head movements and rotational behaviors). After 7 days of modeling, the model group was further divided into four subgroups: the model subgroup, the Tiapride subgroup, the JuA subgroup, and the Tiapride + JuA subgroup (*n* = 6 each). Rats in the Tiapride subgroup were administered 31.25 mg/kg of Tiapride solution and 10 mg/kg of 0.9% NaCl, while rats in the JuA subgroup received 10 mg/kg of JuA solution and 31.25 mg/kg of 0.9% NaCl. Rats in the Tiapride + JuA subgroup were administered a combination of 31.25 mg/kg of Tiapride solution and 10 mg/kg JuA solution. Rats in the model subgroup were given an equivalent volume of 0.9% NaCl. All treatments were administered once daily for 14 consecutive days. The gavage administration volume was set at 10 mL/kg, this dosage was determined based on the conversion factor between humans and animals calculated by body surface area, combined with previous experimental data from our research group (Nair and Jacob 2016).

**FIGURE 1:**
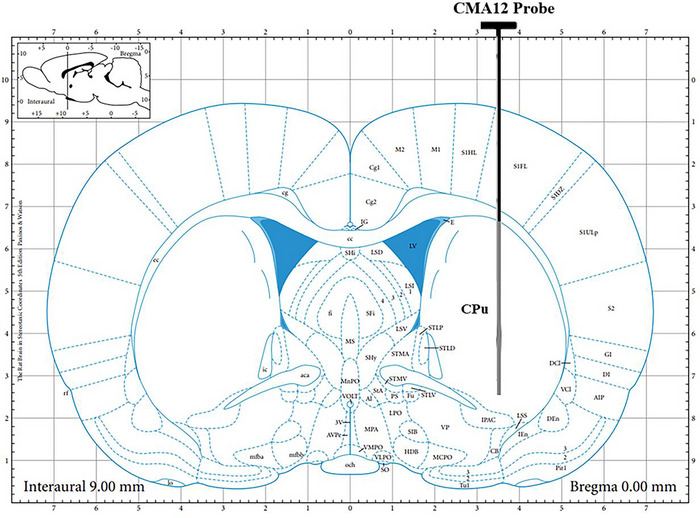
Implantation site of the microdialysis probe in the rat striatal region.

After the completion of drug administration and behavioral observations, rats from each group were anesthetized with 2.0% isoflurane inhalation. Under the guidance of a stereotaxic apparatus, a cannula was implanted into the right CPu of the brain (0.0 mm anterior to the bregma, 3.5 mm lateral to the midline, and 3.4 mm in depth). The CMA12 probe was used with a dialysis membrane length of 4 mm (see Figure 1). The rats were then placed in a freely moving device (33 cm × 36 cm × 40 cm), and the dialysis sampling tubing was connected. The next day, the probe was inserted into the rats while they were awake and freely moving. The rats were perfused with sodium chloride injection at a flow rate of 2.5 µL/min. After of 2‐h equilibration period, dialysate collection began, with one tube collected every 30 min. In this study, to observe the dynamic changes in the levels of Glu and GABA in the CPu of rats after drug administration, a single dose of the drug or saline (for the normal and model groups) was administered via oral gavage following a 60‐min microdialysis period, dialysate was collected after drug administration, and perfusion continued until 180 min. A total of seven tubes (total volume of 450 µL) were collected for the detection of amino acid neurotransmitters using HPLC with fluorescence detection.

After the completion of microdialysis sampling, three rats from each group were randomly selected and euthanized. A portion of the left CPu brain tissue was excised on ice, fixed in 4% paraformaldehyde solution, and subsequently dehydrated, cleared, and embedded in paraffin to create paraffin blocks for immunohistochemical analysis.

### 2.4 Behavioral Tests

Behavioral assessments were conducted on all groups of rats after modeling and at the end of the treatment period (i.e., 2 weeks after the completion of drug administration). Rats were placed in a cage (quiet and light‐avoidant) and allowed to acclimatize for 5 min before scoring began. Two experimenters performed double‐blind observations and evaluated the stereotyped behaviors of the TD rats according to the method described in the referenced literature (Diamond et al. 1982). The scoring criteria were as follows: 0 points: no stereotyped behavior; 1 point: body rotation behavior; 2 points: excessive vertical head and neck movements; 3 points: excessive vertical head and neck movements combined with rotational behavior; 4 points: lateral head movements combined with excessive vertical head and neck movements. Scores were assigned based on the severity of the observed behaviors. Each rat was observed once every 5 min, with each observation lasting 1 min and scored accordingly. A total of 6 observations were made, and the average score was calculated.

### 2.5 Detection of Extracellular Glu and GABA Levels in Rat Striatum by HPLC Method

Dialysate samples from the striatum of rats in each group at various time points were analyzed using HPLC system. The samples underwent pre‐column automatic derivatization with OPA to convert amino acids into fluorescent derivatives. These derivatives were then separated in the chromatographic column and detected by a fluorescence detector (excitation wavelength: 340 nm, emission wavelength: 455 nm). The fluorescence intensity of the amino acid derivatives was measured, and the data were substituted into a standard curve to calculate the concentrations of Glu and GABA in the striatal dialysate at each time point for each group of rats. Derivatization solution preparation: 5 mg of OPA was dissolved in 120 µL of methanol, followed by the addition of 10 µL of β‐mercaptoethanol and 1 mL of borate buffer (0.2 mol/L, pH 9.2), and mixed thoroughly. Mobile phase preparation: Solution A: a mixture of buffer (20 mmol/L sodium acetate solution, pH 7.2), methanol, and tetrahydrofuran in a volume ratio of 400:95:5. Solution B: a mixture of buffer and methanol in a volume ratio of 120:380. Chromatographic conditions: flow rate: 0.8 mL/min; photomultiplier tube: 12; column temperature: 40°C. Gradient elution program: 0–10 min: 0%–63% Solution B; 10–12 min: 63% Solution B; 12–17 min: 100% Solution B; 17–18 min: 100%–0% Solution B; 18–21 min: 0% Solution B.

### 2.6 Immunohistochemical Analysis

Paraffin sections were taken from the striatum of each group of rats, and the slides were baked to dewax in order to perform antigen retrieval to enhance antigen detection. The sections were blocked at room temperature; they were incubated with GLT‐1 primary antibody (1:400) and GLAST primary antibody (1:400) overnight at 4°C. Then, biotin‐labeled secondary antibody was added and incubated at 37°C for 30 min, followed by washing with PBS. DAB chromogen was used for color development until the sections turned brown. The stained sections were washed, counterstained, dehydrated, and cleared, then mounted with neutral gum. For semi‐quantitative analysis, Image Pro Plus 6.0 software was used to randomly select five fields of view under 200× magnification. The integrated optical density and area of the positive regions were measured, and the ratio was calculated accordingly.

### 2.7 Statistical Analysis

All statistical data were expressed as the mean ± standard deviation, and analyses were performed using IBM SPSS 25.0 statistical software. Single‐factor ANOVA was used to compare the differences in experimental data among groups. Normally distributed measurement data were described using mean ± standard deviation ($\;\bar{X}$ ± s) and analyzed using the Shapiro–Wilk test; non‐normally distributed measurement data were described using median (interquartile range) [M (Q1, Q3)] and analyzed using the non‐parametric Kruskal–Wallis test. A *p* value < 0.05 was considered statistically significant.

## 3 Results

### 3.1 Stereotypy Behavior Scores

After modeling, the stereotypy behavior scores of rats in all groups were greater than 1 point, indicating successful model establishment. Post‐administration, there was no statistically significant difference in stereotypy behavior scores between the treatment groups and the model group (*p* > 0.05). After 1 week of administration, the stereotypy behavior scores in the treatment groups were significantly lower than those in the model group (*p* < 0.05). There was no statistically significant difference in stereotypy behavior scores between the JuA group and the Tiapride group (*p* > 0.05). However, the stereotypy behavior scores in the JuA + Tiapride group showed statistically significant differences compared to both the JuA group and the Tiapride group (*p* < 0.05). See Table 1.

**TABLE 1:** Comparison of stereotypy behavior scores among groups [M (Q1, Q3)] (unit: points).

**Group**	**After modeling**	**After 1 week of administration**
Normal group	0	0
Model group	3.00 (3.00, 4.00)	3.00 (2.75, 3.00)
JuA group	3.00 (2.75, 3.00)	2.00 (1.00, 2.25)^[Link]^, ^[Link]^
JuA + Tiapride group	2.00 (2.00, 2.25)	1.00 (0.00, 1.00)^[Link]^, ^[Link]^
Tiapride group	2.00 (1.75, 3.00)	1.00 (0.00, 2.00)^[Link]^, ^[Link]^

^a^Compared with the same group after modeling, *p* < 0.05.

^b^Compared with the model group at the same time point, *p* < 0.05.

### 3.2 Amino Acid Neurotransmitter Levels

At each observation time point, the Glu content in the CPu extracellular fluid of the model group was significantly higher than that of the normal group (*p* < 0.05). The Glu content in all treatment groups was significantly lower than that in the model group (*p* < 0.05), with the JuA + Tiapride group showing the most significant improvement in Glu levels. After a single administration over 60 min, the levels of Glu in all treatment groups showed varying degrees of decrease, with the JuA + Tiapride group experiencing the most significant reduction. At 120, 150, and 180 min, there was no significant difference in Glu levels between the Jua group and the Tiapride group, while significant differences were observed at 0, 30, 60, and 90 min. See Table 2.

**TABLE 2:** Comparison of Glu content in the CPu extracellular fluid among groups ($\;\bar{X}$ ± s) (unit: µg/mL).

**Time**	**Normal group(*n* = 6)**	**Model group(*n* = 6)**	**JuA group(*n* = 6)**	**Tiapride group(*n* = 6)**	**JuA + Tiapride group(*n* = 6)**
0 min	6.73 ± 0.18	18.07 ± 0.89^a^	9.27 ± 0.38^[Link]^, ^[Link]^	8.52 ± 0.30^[Link]^, ^[Link]^	7.60 ± 0.23^[Link]^, ^[Link]^, ^[Link]^
30 min	6.67 ± 0.22	18.05 ± 0.75^a^	9.27 ± 0.45^[Link]^, ^[Link]^	8.49 ± 0.35^[Link]^, ^[Link]^	7.65 ± 0.29^[Link]^, ^[Link]^, ^[Link]^
60 min	6.81 ± 0.23	19.39 ± 0.88^a^	8.98 ± 0.33^[Link]^, ^[Link]^	8.12 ± 0.43^[Link]^, ^[Link]^	7.19 ± 0.38^[Link]^, ^[Link]^, ^[Link]^
90 min	6.68 ± 0.26	21.20 ± 0.59^a^	7.69 ± 0.20^[Link]^, ^[Link]^	7.17 ± 0.28^[Link]^, ^[Link]^	5.54 ± 0.45^[Link]^, ^[Link]^, ^[Link]^
120 min	6.74 ± 0.18	19.51 ± 0.32^a^	6.97 ± 0.20^b^	6.72 ± 0.43^b^	5.42 ± 0.33^[Link]^, ^[Link]^, ^[Link]^
150 min	6.71 ± 0.11	19.31 ± 0.33^a^	6.29 ± 0.15^b^	6.25 ± 0.39^b^	5.29 ± 0.43^[Link]^, ^[Link]^, ^[Link]^
180 min	6.71 ± 0.13	18.74 ± 0.09^a^	6.35 ± 0.10^b^	6.43 ± 0.29^b^	5.62 ± 0.12^[Link]^, ^[Link]^, ^[Link]^

*Note*: A single dose of the drug or saline (for the normal and model groups) was administered after 60‐min microdialysis period, and dialysate was collected after the drug was given.

^a^ The model group compared with the normal group, *p* < 0.05.

^b^ Compared with the model group at the same time point, *p* < 0.05.

^c^ Compared with the JuA group at the same time point, *p* < 0.05.

^d^ Compared with the Tiapride group at the same time point, *p* < 0.05.

The GABA content in the CPu extracellular fluid of the model group was significantly lower than that of the normal group at each observation time point (*p* < 0.05). The GABA content in all treatment groups was significantly higher than that in the model group (*p* < 0.05), with the JuA + Tiapride group showing the most significant improvement. After 60 min, the levels of GABA in all treatment groups showed varying degrees of increase, with the JuA + Tiapride group experiencing the most significant improvement. There was no significant difference in GABA levels between the Jua group and the Tiapride group at each observation time point. At 30 min, there was no significant difference in all treatment groups. See Table 3.

**TABLE 3:** Comparison of GABA content in the CPu extracellular fluid among groups ($\;\bar{X}$ ± s) (unit: µg/mL).

**Time**	**Normal group(*n* = 6)**	**Model group(*n* = 6)**	**JuA group(*n* = 6)**	**Tiapride group(*n* = 6)**	**JuA + Tiapride** **group(*n* = 6)**
0 min	4.87 ± 0.11	3.06 ± 0.10^a^	3.81 ± 0.11^b^	3.77 ± 0.17^b^	4.02 ± 0.14^[Link]^, ^[Link]^, ^[Link]^
30 min	4.86 ± 0.14	3.03 ± 0.12^a^	3.90 ± 0.15^b^	3.81 ± 0.13^b^	4.02 ± 0.19^b^
60 min	4.92 ± 0.13	2.87 ± 0.14^a^	4.19 ± 0.08^b^	4.12 ± 0.10^b^	4.96 ± 0.09^[Link]^, ^[Link]^, ^[Link]^
90 min	4.88 ± 0.14	2.97 ± 0.10^a^	4.38 ± 0.10^b^	4.30 ± 0.13^b^	5.22 ± 0.13^[Link]^, ^[Link]^, ^[Link]^
120 min	4.90 ± 0.11	3.01 ± 0.14^a^	4.56 ± 0.17^b^	4.49 ± 0.13^b^	5.43 ± 0.18^[Link]^, ^[Link]^, ^[Link]^
150 min	4.89 ± 0.13	3.00 ± 0.10^a^	4.67 ± 0.20^b^	4.57 ± 0.15^b^	5.62 ± 0.30^[Link]^, ^[Link]^, ^[Link]^
180 min	4.90 ± 0.16	2.98 ± 0.04^a^	4.71 ± 0.20^b^	4.62 ± 0.12^b^	5.64 ± 0.36^[Link]^, ^[Link]^, ^[Link]^

*Note*: A single dose of the drug or saline (for the normal and model groups) was administered after 60‐min microdialysis period, dialysate was collected after the drug was given.

^a^ The model group compared with the normal group, *p* < 0.05.

^b^ Compared with the model group at the same time point, *p* < 0.05.

^c^ Compared with the JuA group at the same time point, *p* < 0.05.

^d^ Compared with the Tiapride group at the same time point, *p* < 0.05.

### 3.3 Comparison of GLAST and GLT‐1 Protein Expression in CPu Tissue

**FIGURE 2:**
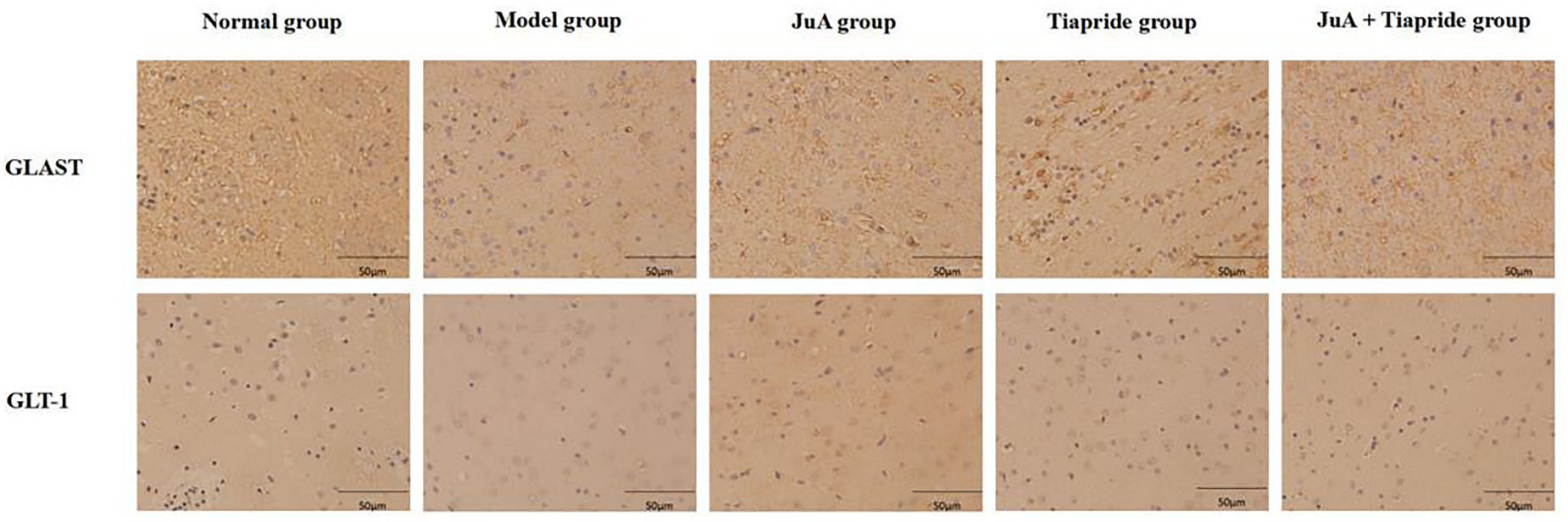
Protein expression of GLAST and GLT‐1 in the striatum of rats (immunohistochemical method, × 200).

The protein expression of GLAST and GLT‐1 in the CPu tissue of the model group rats was significantly lower than that of the normal group (*p* < 0.05); the protein expression in each treatment group was significantly higher than that in the model group (*p* < 0.05). Among them, the protein expression in the JuA + Tiapride group was significantly higher than that in the Jua group and the Tiapride group (*p* < 0.05), while there was no significant difference in protein expression between the Jua group and the Tiapride group (*p* > 0.05). See Figure 2, and Table 4.

**TABLE 4:** Comparison of GLAST and GLT‐1 protein expression in CPu tissue among different groups of rats ($\;\bar{X}$ ± s).

**Group**	**Number**	**GLAST**	**GLT‐1**
Normal group	3	0.388 ± 0.003	0.283 ± 0.001
Model group	3	0.308 ± 0.002^a^	0.245 ± 0.002^a^
JuA group	3	0.370 ± 0.001^b^	0.265 ± 0.003^b^
Tiapride group	3	0.366 ± 0.001^b^	0.259 ± 0.001^b^
JuA + Tiapride group	3	0.380 ± 0.002^[Link]^, ^[Link]^, ^[Link]^	0.283 ± 0.003^[Link]^, ^[Link]^, ^[Link]^

^a^ The model group compared with the normal group, *p < 0.05.

^b^ Compared with the model group at the same time point, *p* < 0.05.

^c^ Compared with the JuA group at the same time point, *p* < 0.05.

^d^ Compared with the Tiapride group at the same time point, ☆*p* < 0.05.

## 4 Discussion

Glu, an excitatory neurotransmitter, exerts its stimulatory effects by binding to receptors on postsynaptic neurons; however, its accumulation in the synaptic cleft can result in neurotoxicity (Abe et al. 2023). It has been reported that an imbalance in Glu levels within the brains of individuals with TD results in neuronal hyperexcitability. Animal studies have confirmed that reducing Glu levels in the brain significantly ameliorates tic‐like behaviors, suggesting that Glu dysregulation is a critical factor in the onset of TD (Naaijen et al. 2016; Xi et al. 2022). Glu exerts its effects by activating NMDA receptors, triggering a substantial influx of Ca^2+^, which subsequently causes damage to dopaminergic neurons (Yang et al. 2022; Kanaan et al. 2017). GABA is an inhibitory neurotransmitter that is widely distributed throughout the CNS; it functions by binding to the GABA(A) receptor (GABAAR) on postsynaptic neurons, modulating ion channels to promote membrane hyperpolarization. This action reduces neuronal excitability and plays a crucial role in regulating mood and excitatory behaviors in humans (Sears and Hewett 2021; McArdle et al. 2024). It has been reported that lower levels of GABA in the primary sensorimotor cortex, supplementary motor area, and insular cortex are associated with more severe and frequent premonitory urges in TD (He et al. 2022). In addition, Glu and GABA interact with other neurotransmitters, such as serotonin (5‐HT), collectively influencing dopaminergic neurons in the brain and contributing to the pathogenesis of TD (Franco et al. 2021; Najm Al‐Halboosi et al. 2023). Currently, Tiapride, a selective dopamine D2 receptor antagonist, is one of the most effective medications for treating TD (Fekete et al. 2021). However, it still encounters challenges, including inadequate efficacy in some patients, significant side effects, and suboptimal long‐term outcomes (Billnitzer and Jankovic 2020). Therefore, the search for safer and more effective interventions remains an urgent priority. JuA is one of the primary active components of *Semen Ziziphi Spinosae*, a traditional Chinese medicinal herb that consists of the dried seeds of *Ziziphus jujuba*. It is known for its pharmacological effects, particularly in the central nervous system, including sedative, anxiolytic, and neuroprotective properties (Bae et al. 2023; Hua et al. 2022). JuA is being investigated for its potential therapeutic effects on TD or related neurological conditions, but the mechanism is not clear (Fan, Hao et al. 2022).

The findings of this study indicate that, compared to the normal group, the model group exhibited significantly increased extracellular Glu levels and markedly decreased GABA levels in the CPu. Additionally, the stereotypical behavior scores in the model group rats were significantly higher. These results suggest that alterations in Glu and GABA levels may be associated with the manifestation of stereotypical behaviors in TD rats. In the administration groups, the extracellular Glu levels in the CPu of rats were significantly lower at all time points compared to the normal group, while GABA levels were significantly higher. Concurrently, the stereotypical behavior scores in these rats were markedly lower than those in the model group. These findings suggest that Jua and Tiapride may ameliorate the stereotypical behaviors in TD rats by facilitating the transport of Glu in the CPu extracellular fluid, reducing Glu accumulation, and increasing GABA levels, thereby mitigating the neurotoxicity induced by IDPN. In this study, to observe the dynamic changes in Glu and GABA levels in the CPu of rats after drug administration, microdialysis was performed for 60 min followed by a single oral administration of either the drug or saline (normal group, model group). It was found that after saline administration, the model group exhibited a trend of increasing Glu levels and decreasing GABA levels, whereas the normal group maintained stable levels of these indicators, however, these changes did not reach statistical significance. In contrast, all treatment groups showed a trend of decreasing Glu levels and increasing GABA levels. These results suggest that oral administration may stimulate changes in Glu and GABA levels in the extracellular fluid of the CPu in TD rats. The model group demonstrated poor regulatory capacity for these changes, while all treatment groups exhibited some degree of regulatory ability. However, further experiments involving longer dialysis collection periods are necessary to validate these findings. Furthermore, this article has some limitations. First, postoperative drowsiness, alterations in motor behavior, sleep disturbances, and neuroinflammatory responses may all influence treatment outcomes. Second, this study only included male subjects, there were no female participants. Gender differences in the response to JuA during TD treatment may exist (Nordstrom and Burton 2002), future research could explore this further.

Excessive synaptic Glu levels can lead to overstimulation of postsynaptic Glu receptors, resulting in excitotoxicity and neuronal damage (Verma et al. 2022; Gorska and Eugenin 2020). Research suggests that unhealthy dietary intake may influence this process by triggering microglia‐mediated neuroinflammation (Dar 2024; Fan, Bian et al. 2022). It has been discovered that substances such as insulin‐like growth factor‐1 (IGF‐1) and prolactin, which are involved in normal growth and development, exert neurotrophic and neuroprotective effects, these substances play a crucial role in mitigating excitotoxicity and reducing neuronal damage in neurological disorders (Ge et al. 2022; Rodriguez‐Chavez et al. 2021; Trinh et al. 2021). Excitatory amino acid transporters (EAATs) are responsible for the transport of excitatory amino acids and are associated with various neuropsychiatric disorders (Gan et al. 2024; Alijanpour et al. 2023; Iovino et al. 2020). The primary Glu transporters in the CNS are excitatory amino acid transporter‐1 (EAAT1) and excitatory amino acid transporter‐2 (EAAT2). GLAST and GLT‐1 are the rodent homologs of EAAT1 and EAAT2, respectively (Jiménez et al. 2014). Studies have shown that inhibiting GLAST increases extracellular Glu levels, leading to excitotoxic neuronal death in mice (Maragakis and Rothstein 2004). Similarly, GLT‐1 knockout rats exhibit spontaneous seizures and neuronal loss, whereas functional GLT‐1 can prevent post‐traumatic epilepsy in rat models of traumatic brain injury (Pajarillo et al. 2019). The results of this study indicate that the expression of GLT‐1 and GLAST proteins in the CPu tissue of the model group rats was significantly lower compared to the normal group. In contrast, the expression levels of GLT‐1 and GLAST proteins in the CPu tissue of the drug‐administered groups were markedly increased. These findings suggest that JuA and Tiapride may ameliorate stereotypical behaviors in TD rats by upregulating the expression of GLT‐1 and GLAST in the CPu tissue, thereby helping to maintain relatively stable levels of neurotransmitters.

In summary, this study utilized microdialysis technology to assess the temporal changes in Glu and GABA levels in the extracellular fluid of the CPu in TD rats. It also compared the effects of three treatment regimens on the levels of these two neurotransmitters. The research highlights the therapeutic potential of JuA for TD and reveals that the combined use of Tiapride and JuA offers superior improvement in neurotransmitter levels in the CPu extracellular fluid and better control of tic symptoms in model rats compared to either drug used alone. This effect may be related to the modulation of GLT‐1 and GLAST protein expression levels. We strongly recommend further in‐depth research into JuA‐based treatment strategies for TD.

## Author Contributions

F.F. and S.Z. designed the research study and performed the research. F.H., H.W., and M.Z. provided help and advice on the manuscript. F.F. wrote the manuscript. All authors contributed to editorial changes in the manuscript. All authors read and approved the final manuscript.

## Funding

This research was funded by the Fundamental Research Funds for the Central Public Welfare Research Institutes, Beijing, China (Nos. ZZ15‐XY‐PT‐03 and ZZ17‐XRZ‐043).

## Ethics Statement

All experimental protocols were approved by the Animal Studies Ethics Committee of Medical Experiment Center, China Academy of Chinese Medical Sciences (approval number, ERCCACMS21‐2111‐16).

## Conflicts of Interest

The authors declare no conflicts of interest.

## Supporting information


**Supplementary Materials**: brb371041‐sup‐0001‐SuppMatt.docx

**Supplementary Materials**: brb371041‐sup‐0002‐SuppMatt.xlsx

**Supplementary Materials**: brb371041‐sup‐0003‐SuppMatt.xlsx

## Data Availability

The data sets used and analyzed during the current study are available from the corresponding author on reasonable request.
