# Supplementary material for: Modulatory Effects of Jujuboside A on Amino Acid Neurotransmitter Profiles in Tic Disorder
Source: Brain Behav. 2025 Nov 11;15(11):e71041. doi: 10.1002/brb3.71041 (PMC12606026; doi:10.1002/brb3.71041)
Supplement: Supplementary file 1 — Supplementary Materials: brb371041‐sup‐0001‐SuppMatt.docx [file BRB3-15-e71041-s003.docx]

**Supplemental-Main Instruments**

68025 Brain Stereotaxic Apparatus, 90-102 Handheld Cranial Drill, Shenzhen RWD Life Science Co., Ltd.; Microdialysis System, including CMA120 Awake Animal Activity Device, Harvard PUMP11 ELITE Microinjection Pump, CMA/470 Low-Temperature Automatic Sample Collector, CMA/12 Probe (membrane length 4 mm, membrane diameter 0.5 mm, cutoff 6 kD), and cannula, CMA, Sweden; S-501 High-Performance Liquid Chromatography System, SYKAM, Germany; RF-20AXL Fluorescence Detector, Shimadzu, Japan; Eclipse AAA Fluorescence Detection Column (4.6 mm × 150 mm, 5 μm), Agilent, USA; arium 61316 Reverse Osmosis Pure Water System, BP211D One-Hundred-Thousandth Electronic Balance, PB-21 pH Meter, Sartorius, Germany; Milli-Q Ultrapure Water System, Millipore, USA; 0.22 μm Microporous Membranes for Aqueous and Organic Phases, Tianjin Jinteng Experimental Equipment Co., Ltd.
